# Ectopic expression of a novel cold-resistance protein 1 from *Brassica oleracea* promotes tolerance to chilling stress in transgenic tomato

**DOI:** 10.1038/s41598-021-96102-3

**Published:** 2021-08-16

**Authors:** Umer Majeed Wani, Sheikh Tahir Majeed, Vaseem Raja, Zubair Ahmad Wani, Nelofer Jan, Khursid Iqbal Andrabi, Riffat John

**Affiliations:** 1 Plant Molecular Biology Lab, Department of Botany, University of Kashmir, Srinagar, Kashmir 190 006 India; 2 Department of Biotechnology, University of Kashmir, Srinagar, India

**Keywords:** Plant molecular biology, Plant physiology, Plant stress responses

## Abstract

Cold stress is considered as one of the major environmental factors that adversely affects the plant growth and distribution. Therefore, there arises an immediate need to cultivate effective strategies aimed at developing stress-tolerant crops that would boost the production and minimise the risks associated with cold stress. In this study, a novel cold-responsive protein1 (*BoCRP1*) isolated from *Brassica oleracea* was ectopically expressed in a cold susceptible tomato genotype Shalimar 1 and its function was investigated in response to chilling stress. *BoCRP1* was constitutively expressed in all the tissues of *B. oleracea* including leaf, root and stem. However, its expression was found to be significantly increased in response to cold stress. Moreover, transgenic tomato plants expressing *BoCRP1* exhibited increased tolerance to chilling stress (4 °C) with an overall improved rate of seed germination, increased root length, reduced membrane damage and increased accumulation of osmoprotectants. Furthermore, we observed increased transcript levels of stress responsive genes and enhanced accumulation of reactive oxygen species scavenging enzymes in transgenic plants on exposure to chilling stress. Taken together, these results strongly suggest that *BoCRP1* is a promising candidate gene to improve the cold stress tolerance in tomato.

## Introduction

Cold stress is considered as a pivotal component of environmental stress as it is one of the major contributing factors that severely affect the plant growth and productivity across the globe. Since plants are sessile in nature, they are immobile and can respond to the cold stress only by changing the expression pattern of specific stress-related genes^1^. A microarray analysis by Seki, et al.^2^ demonstrate that under cold stress conditions a significant number of stress responsive genes get differentially expressed. There are a range of stress responsive genes and transcription factors identified and characterised hitherto across different plant species^3,4^ The specific interaction between a C-repeat/dehydration response elements (CRT/DRE) present in the promoter region of cold regulated (COR) genes with CRT/DRE binding factors (CBFs) regulates the expression pattern of many downstream genes most of which are stress responsive genes with a role in cold tolerance, salinity and drought^5–8^. The involvement of CBF proteins (CBF1, CBF2 or CBF3) in imparting cold tolerance is strengthened by the fact that transgenic lines constitutively over-expressing CBF proteins have concomitant expression of many cold regulated genes and subsequently improved tolerance to cold^9–12^ such that down-regulation of both CBF1 and CBF3 resulted in a decline in expression of CBF regulatory genes in Arabidopsis which was also marked by decreased tolerance to cold^13^. While some of the COR proteins have role in imparting tolerance to cold stress by cryoprotection has been established. However, much needs to be studied vis-à-vis role of CBF regulon encoded proteins in cold tolerance and the subject remains open for thorough and elaborate research. COR genes are classified and grouped into four gene families such that each family is constituted of two members. These gene families include KIN (cold-induced), RD (responsive to desiccation), LTI (low temperature-induced) and ERD (early dehydration-inducible), within a particular family of genes, each gene is in tandem association with the other member in the genome^14^. The CRISPR/Cas9 generated mutants of CBF including single, double and triple offered enough evidence to state that CBF genes regulates the expression of 414 downstream COR genes, besides the rate of seed germination was significantly lower in case of CBF mutants compared to WT^15^. Higher transcript accumulation of COR/ late embryogenesis abundant (LEA) genes has been associated with higher germination index in common wheat^16^. Researchers have identified vast number of cold-responsive genes in Arabidopsis and other plant which include KIN1, KIN2, RD29A, RD29B, DREBs and DELLA^17–22^. These cold responsive genes (KIN1, KIN2, COR15A, COR15B RD29A and RD29B,) are present as on the same chromosome as tandem sequences^18,23,24^. Kin1 and Kin2 are coordinately regulated in cold. Kin2 mRNA is accumulated to a higher level during cold acclimation^18^. Globally tomato (*lycopersicum esculentum*) is an economically important crop that is susceptible to a series of abiotic stresses especially cold and drought. It is well known that the cold and drought stress negatively affects the plant growth which has a significant impact on the tomato production^25^. Therefore, effective strategies need to be devised for the development of cold resistant tomato varieties to meet the growing demand and minimize the economic losses worldwide.

The fundamental interest in understanding the molecular mechanisms governing the cold stress stems from the thought that such an insight will aid in devising new strategies aimed at engineering the agronomically important crops for enhanced tolerance to cold. In the last few decades, a lot of strategies involving genetics, functional genomics, physiology and biochemistry have been employed to understand the response of plants to different environmental stresses such drought, cold and salinity. Previously, we have performed proteomic study to compare the gene expression analysis in Brassica oleracea var. “capitata” under both normal (25 °C) and cold conditions (4 °C). Under cold conditions, a novel low molecular weight protein called cold induced protein 1 (*BoCRP1*) was found to be highly induced. Here, we focus on the functional aspect of *BoCRP1* whose expression analysis indicates that *BoCRP1* is constitutively expressed in different tissue samples of *B. oleracea* variety *capitata* and rapidly induced under cold stress conditions. This *BoCRP*1 protein is a member of Kin family and shows about 90% homology with other members of KIN gene family (KIN1 and Kin2). To identify the possible role of *BoCRP1* gene, we looked for the genome data and found that two Kin sequences from *A. thaliana* Kin1 (At5g15960) and Kin2 (At5g15970) show high level of homology to *BoCRP1* gene. There are a number of highly similar DNA sequences in related organisms. The one coding identical protein sequences in other *B. oleracea* species are annotated as cold-resistant proteins, KIN1 and KIN2. To explore the possible mechanism of cold stress we tried to characterize the function of *BoCRP1* by overexpressing *BoCRP1* under stress inducible promoter AtRd29A in cold susceptible tomato. Our findings suggest that overexpressing *BoCRP1* in cold susceptible tomato renders it tolerant to cold stress.

## Results

### Isolation and Sequence Analysis of *BoCRP1* gene

**Figure 1:**
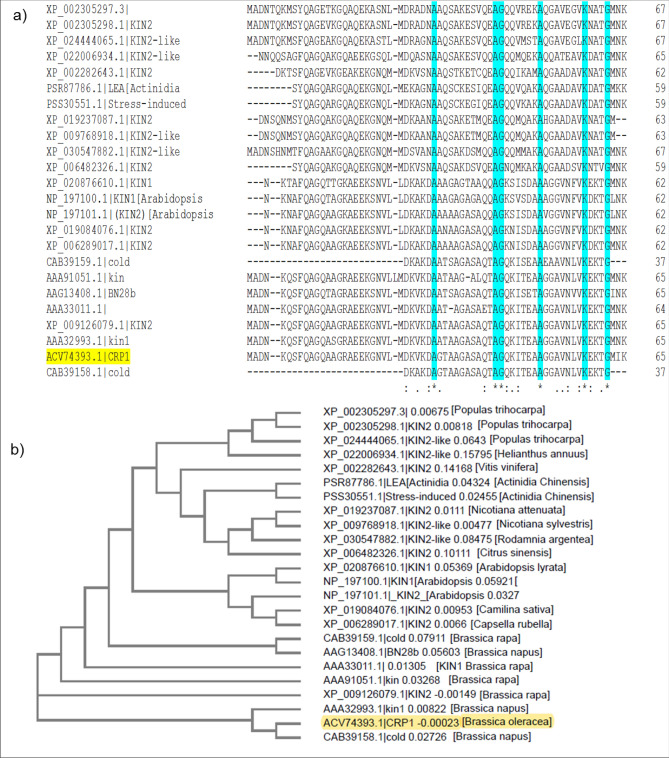
Sequence alignment and phylogeny of BoCRP1 homologues. (**a**) Multiple sequence alignment of amino acid homologues. Identical conserved consensus amino acids are indicated by asterisks, whereas conserved substitutions are indicated by colons and periods. (**b**) A phylogenetic tree was constructed using ClustalW2 program by neighbor-joining method.

The CDS of of *BoCRP1 (*Accession no*.GQ461800.1*) consists of 198 bps which encodes a low molecular weight protein of 65amino acids with a molecular weight of 6.5 KDa, PI approaching to 9.1. Various sequence alignment tools revealed a strong homology between *BoCRP1* protein and Kin proteins of *B. napus*, *B. rapa* and *A. thaliana* annotated cold-resistant proteins (Fig. 1a). On performing the phylogenetic analysis, it was clear that *BoCRP1* has a close semblance to homologue from *A. thaliana, B. rapa and B. napus*, (Fig. 1b). As shown in Fig. 1, LEA proteins are also similar stress related proteins which are homologous to *BoCRP1* protein. However, *BoCRP1* is closer to Kin1/Kin2 of Arabidopsis than to LEA-related protein from Actinidia chinensis. We found that the closest relative plant to *B. oleracea* on STRING database is *Brassica rapa*. It has a close relative (ortholog) of *BoCRP1*. In *B. rapa,* the gene (Bra008661) is connected to BRA000263 (COR15B Cold regulated gene), which suggests that Bra008661 (and hence *BoCRP1* too is involved in the cold response. Homology models showed a folded alpha-helix structure of *BoCRP1* similar to that of KIN2 of *A. thaliana*^26^. All the above findings strongly advocate the involvement of *BoCRP1* protein in cold resistance.

### Transcript analysis of *BoCRP1* in *B. oleracea*

**Figure 2:**
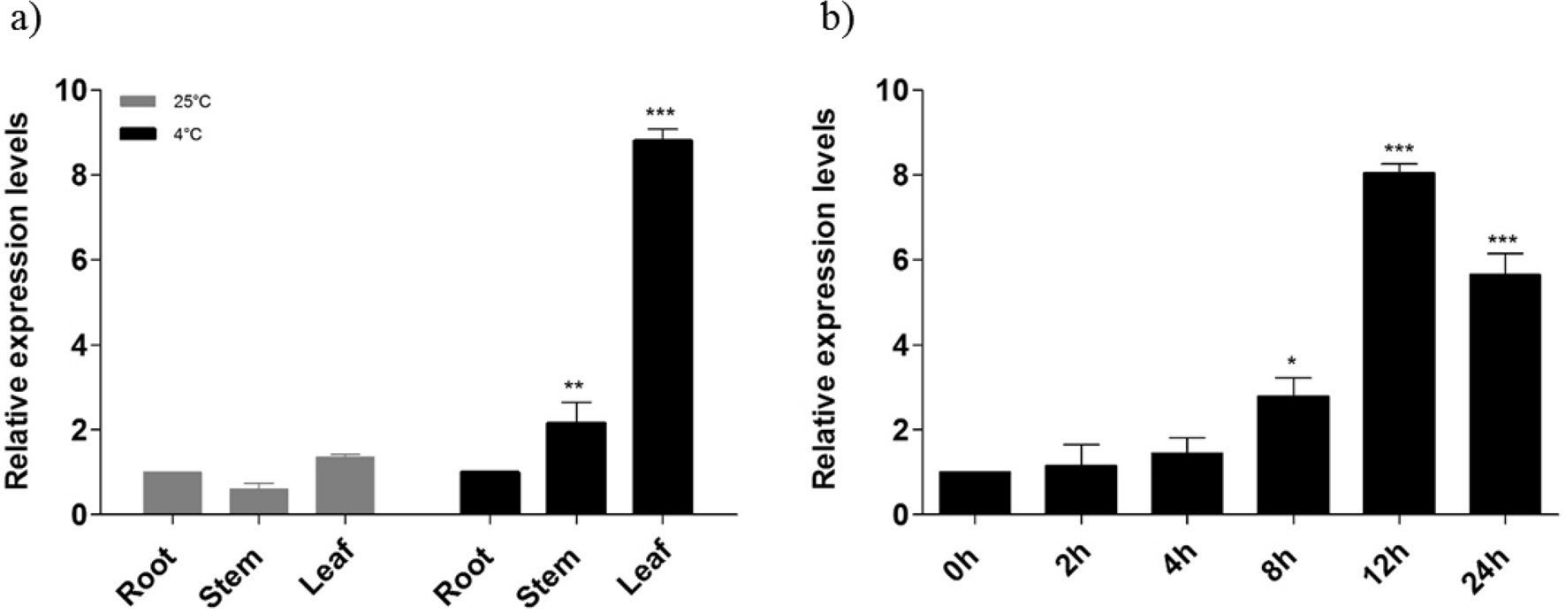
Expression pattern BoCRP1 in *Brassica oleracea* (**a**) Tissue specific expression of BoCRP1 in root, stem and leaf under both normal (25 °C) and cold (4 °C) after 12 h. (**b**) qPCR data showing time course expression of BoCRP1 under cold stress conditions (4 °C) for 24 h in *Brassica oleracea*. The bars indicates standard deviation (SD) and Asterisks represents significant differences between wild-type and transgenic lines. *P < 0.05; **P < 0.01; ***P < 0.001 Tukey's Multiple Comparison Test.

To examine the tissue specific mRNA levels of *BoCRP1* in *B. oleracea* var. *capitata*, plants were exposed to cold (4 °C) for varying time periods. Expression studies indicated that the *BoCRP1* transcript levels were highly up-regulated and reached a maximum up to eightfold after 12 h. After that, the expression shows a gradual decline (Fig. 2b). These results strongly suggest that *BoCRP1* plays an important role by offering an early response to cold stress.

Tissue-specific expression analysis exhibited enhanced mRNA levels of *BoCRP1* in the leaf tissues when exposed to cold. However, comparatively lower levels of *BoCRP1*were observed in the stem and the root tissues under cold compared to normal conditions (Fig. 2a).

### Transformation and molecular Analysis

**Figure 3:**
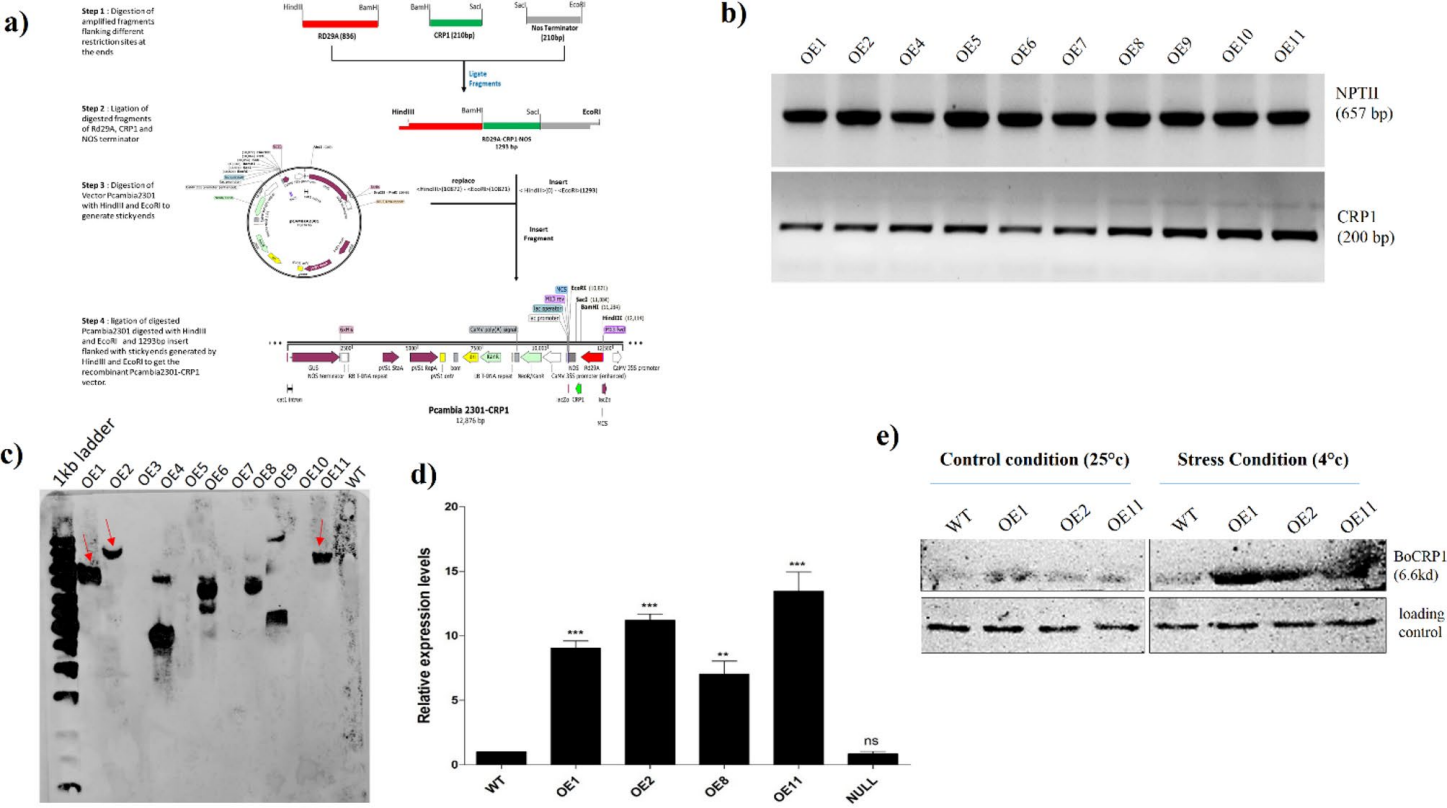
Cloning and molecular analysis of transgenic plants. (**a**) Schematic representation cloning strategy of *BoCRP1* gene (Green) under stress inducible promoter Rd29A (Red) in plant binary vector Pcambia2301 (**b**) screening of kanamycin positive lines by PCR, using NPTII and CRP1 specific primers. (**c**) Southern blot analysis of PCR positive transgenic lines for single copy insertion lines. The dioxyginin labelled NPTII probe were used to check the insertion and copy number of T-DNA regions within the plant genome. Only four transgenic lines were found to contain single copy T-DNA region. (**d**) Expression pattern of BoCRP1 transcript in four different transgenic lines after cold stress. qRT-PCR analysis depicts that out of four putative transgenic lines , expression of *BoCRP1* is higher in OE1, OE2 and OE11. (**e**) Western blot indicating the expression of BoCRP1 protein in different overexpression lines under both normal and cold conditions using GAPDH as loading control. Asterisks indicate significant differences between wild-type (WT) and transgenic lines). *P < 0.05; **P < 0.01; ***P < 0.001 Tukey's Multiple Comparison Test.

To validate and characterize the function of *BoCRP1* in cold susceptible tomato variety “Shalimar 1” the plant binary vector pcambia2301 was selected to clone the entire ORF of *BoCRP1* gene under a stress-inducible promoter of Rd29A (Fig. 3a).The transformation of the recombinant vector was executed in tomato cultivator Shalimar 1 (Fig. S1a-d) and obtained 20 independent kanamycin-resistant tomato lines (T_0_ generation).

Using NPTII and *BoCRP1* specific primer sequences, a total of 10 transgenic lines were identified (Fig. 3b). Among them, four stably transgenic lines, OE1, OE2, OE8 and OE11 were confirmed to contain single-copy insertion and segregated in 3:1 ratio for antibiotic selection possibly due to single T-DNA transfer (Fig. 3c). In order to assess the transgene expression, q-PCR was performed to analyse different the transgenic lines. We obtained three putative independent transgenic lines OE1, OE2 and OE11 with significant proportion of transgene expression under cold stress (Fig. 3d). Furthermore, to confirm the expression of *BoCRP1* in selected transgenic lines viz OE1, OE2 and OE3 at translational level, western blotting was performed. As seen in Fig. 3e, induced *BoCRP1* expression was observed in all the three transgenic lines at 4 °C compared to WT. These selected transgenic lines were further allowed to grown for 2 to 3 generations to obtain the homozygous (T3) lines.

### Overexpression of *BoCRP1* in tomato improved the seedling growth and seed germination

**Figure 4:**
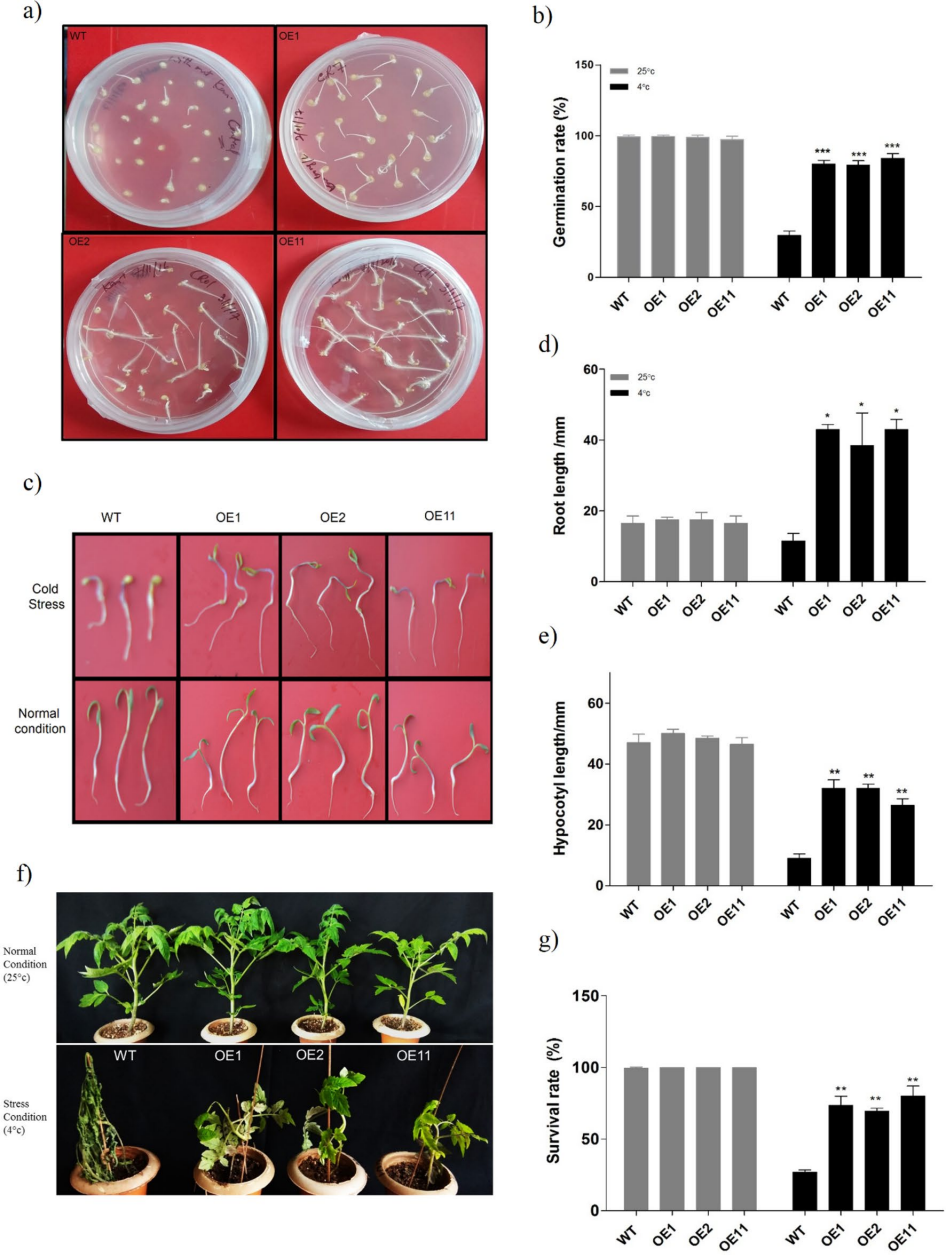
Low temperature induced stress tolerance in transgenic tomato plants over- expressing the *BoCRP1* gene under cold stress. (**a**) Rate of germination of transgenic and wild-type seeds under cold stress (4 °C). The rate of seed germination were calculated at the times when root emerges from the seed and photograph was taken after 2 weeks at 4 °C. (**b**) Graphical representation of the mean of three independent experiments. In each individual experiment the rate of seed germination was averaged from a total of 26 seeds. (**c**) Root and hypocotyl development in early seeding of wild-type and transgenic plantlets under normal and stress conditions. (**d**, **e**) Graphical representation of average root and hypocotyl length of transgenic and wild-type seedlings. (**f**) Phenotypic evaluation of tomato plants under Ambient Temperatures (25 °C) after recovery for 2 days post cold stress (4 °C) treatment for 4 days. The photograph was taken after 2 days of recovery. (**g**) Represents the survival percentage of WT, OE1, OE2 and OE11 plants during recovery after cold treatment for 4 days. Data represents the average of 3 independent experiments. A total of 100 plants of each line including wild-type were used for the experiment. The bars indicates standard deviation (SD) and Asterisks represents significant differences between wild-type and transgenic lines. *P < 0.05; **P < 0.01; ***P < 0.001 Tukey's Multiple Comparison Test.

To assess the cold stress tolerance, it was imperative for us to study the germination rate and seedling growth in both transgenic lines as well as in WT under normal (25 °C) and cold conditions (4 °C). At 25 °C, we observed a similar germination rate both in WT as well as in transgenic lines, confirming that both the seeds are 100% viable. However, the rate of seed germination was significantly up in transgenic lines relative to the WT under chilling stress (4 °C) (Fig. 4a). The germination rate of transgenic lines OE1, OE2, and OE11 was approximately 80%, 79%, and 84% respectively compared to about 30% in WT under stress conditions (Fig. 4b).

Furthermore, to understand whether *BoCRP1* over-expression influence the seedling growth in transgenic lines, seedlings after germination were placed at10 °C for a period of 2 weeks, following which the hypocotyl length along with main root length was measured with a ruler. In *BoCRP1* expressing plants, no obvious difference in the root and hypocotyl length were observed compared to wild-type at 25 °C. Interestingly, at 10 °C both the root as well as hypocotyl length was suppressed in WT as compared to *BoCRP1* expressing lines (Fig. 4c), which otherwise displayed a marked increase in root and hypocotyl length (Fig. 4d,e). The above results demonstrate that the cold-induced expression of *BoCRP1* can modulate the ability of a plant to resist low temperatures as was observed in early seedling and germination stage of transgenic tomato.

### Ectopic expression of *BoCRP1* in transgenic tomato enhances tolerance to cold

To further understand the role of *BoCRP1*, we investigated the functional significance and physiological effect of *BoCRP1* under chilling stress (4 °C) in both WT as well as transgenic lines. For this we incubated plants at 4 °C in the growth chamber for 4 days and then shifted to recovery at 25 °C for 2 to 3 days. At ambient temperatures (25 °C), we observed no significant differences at any stage, neither in transgenic lines nor in WT tomato plants (Fig. 4f). However, transgenic lines showed a survival rate of about 73% (OE1), 68% (OE2), and 80% (OE11), respectively, while only 26% of the WT plants survived during recovery (Fig. 4e), suggesting an increased tolerance in *BoCRP1* expression lines under the chilling stress.

### *BoCRP1* transgenic plants accumulate increased osmoprotectants under cold conditions

**Figure 5:**
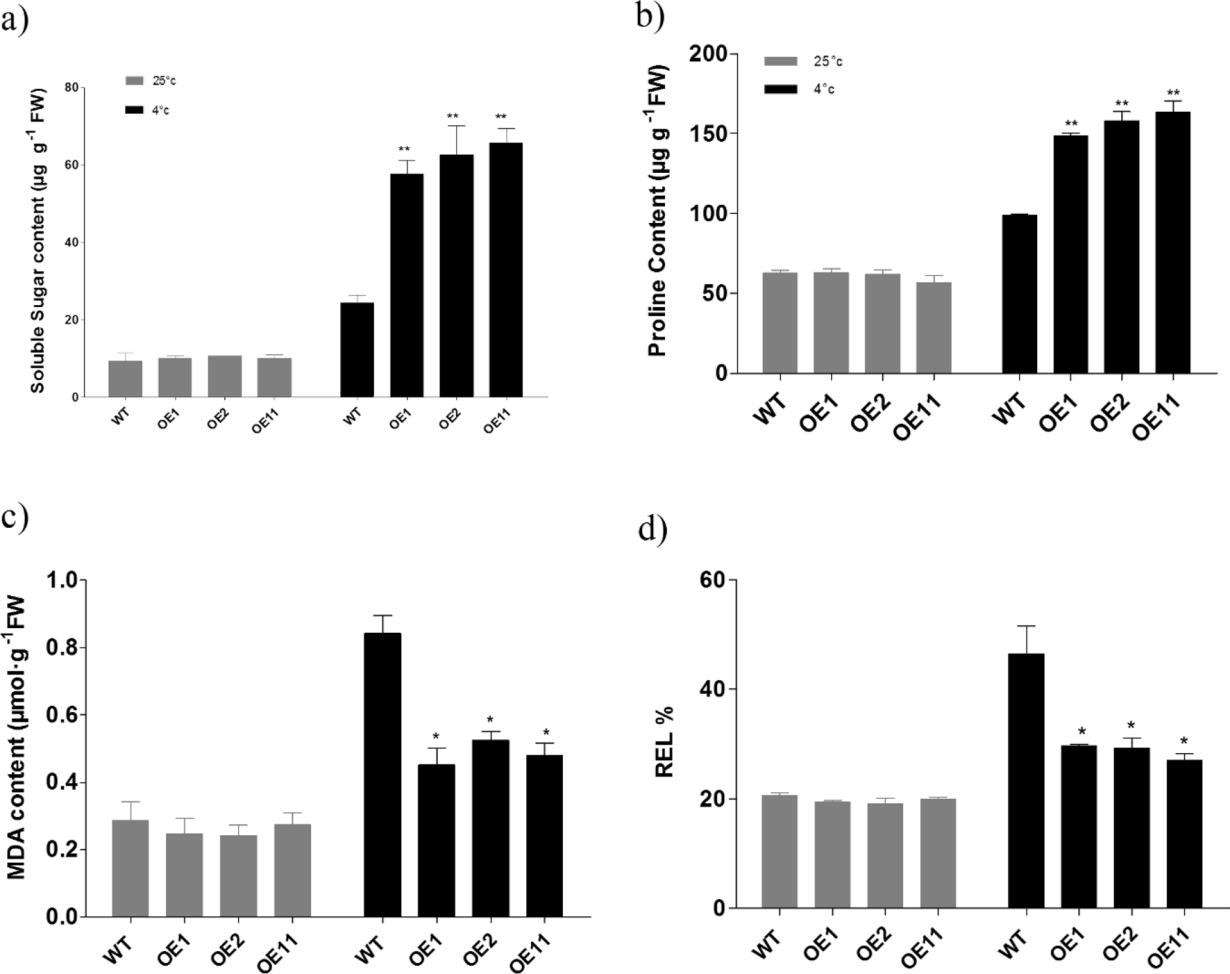
Low temperature induced accumulation of osmoprotactants and Evaluation of membrane damage in WT and transgenic lines (**a**) represents soluble sugar accumulation by transgenic and wild-type tomato (**b**) proline accumulation by WT and BoCRP1 expressing tomato plants at ambient temperatures and after being treated cold stress (4 °C) for 3 days. (**c**) MDA content (**d**) REL. The data represents the mean ± SD of three Biological replicates Asterisks represents the statistically significant differences between WT and *BoCRP1* expressing lines. *P < 0.05;**P < 0.01; ***P < 0.001 Tukey's Multiple Comparison Test.

Under normal growth conditions, the WT and *BoCRP1* expressing lines showed similar content of osmoprotectants **(**proline and soluble sugar). However, we observed significant accumulation of both the osmoprotectants at 4 °C in *BoCRP1* lines compared to WT. Further, soluble sugar content increased by 2.4, 2.6 & 2.7 fold in transgenic line OE1, OE2 and OE11 respectively compared to WT under cold stress (Fig. 5a). Similarly, proline content enhanced by 1.5 fold in OE1, 1.6 fold in OE2 and OE11 respectively, compared to WT (Fig. 5b).

### Overexpression of *BoCRP1* improves membrane stability in transgenic lines

In the context of Abiotic stress (cold, drought and salt) the level of malondialdehyde (MDA) and and relative electrolyte leakage (REL) is considered one of the vital parameter in evaluating the effect on lipid peroxidation and cytomembrane penetrability^27^. While evaluating the levels of MDA and REL in wild-type and *BoCRP1* expressing plants, we observed no obvious differences in MDA and REL content in wild-type and *BoCRP1* lines under control conditions (25 °C) (Fig. 5c,d). However, under cold conditions both the WT and *BoCRP1* lines displayed an increase in REL and MDA content relative to the controls grown under ambient temperatures (25 °C). Cold stressed transgenic seedlings showed significantly reduced content of MDA and REL levels. Above results concluded that in WT plants cold treatment induces 2.9 fold increase in MDA content and only 1.8, 2.1 and 1.7 fold increase in OE1, OE2 and OE11 lines respectively (Fig. 5c) and the REL increased by almost 2.3 fold in WT and only 1.5, 1.5 and 1.3 fold increase in OE1, OE2 and OE3 respectively (Fig. 5d).

### Overexpression of *BoCRP1* improves the ROS scavenging capacity to enhance tolerance to cold stress.

**Figure 6:**
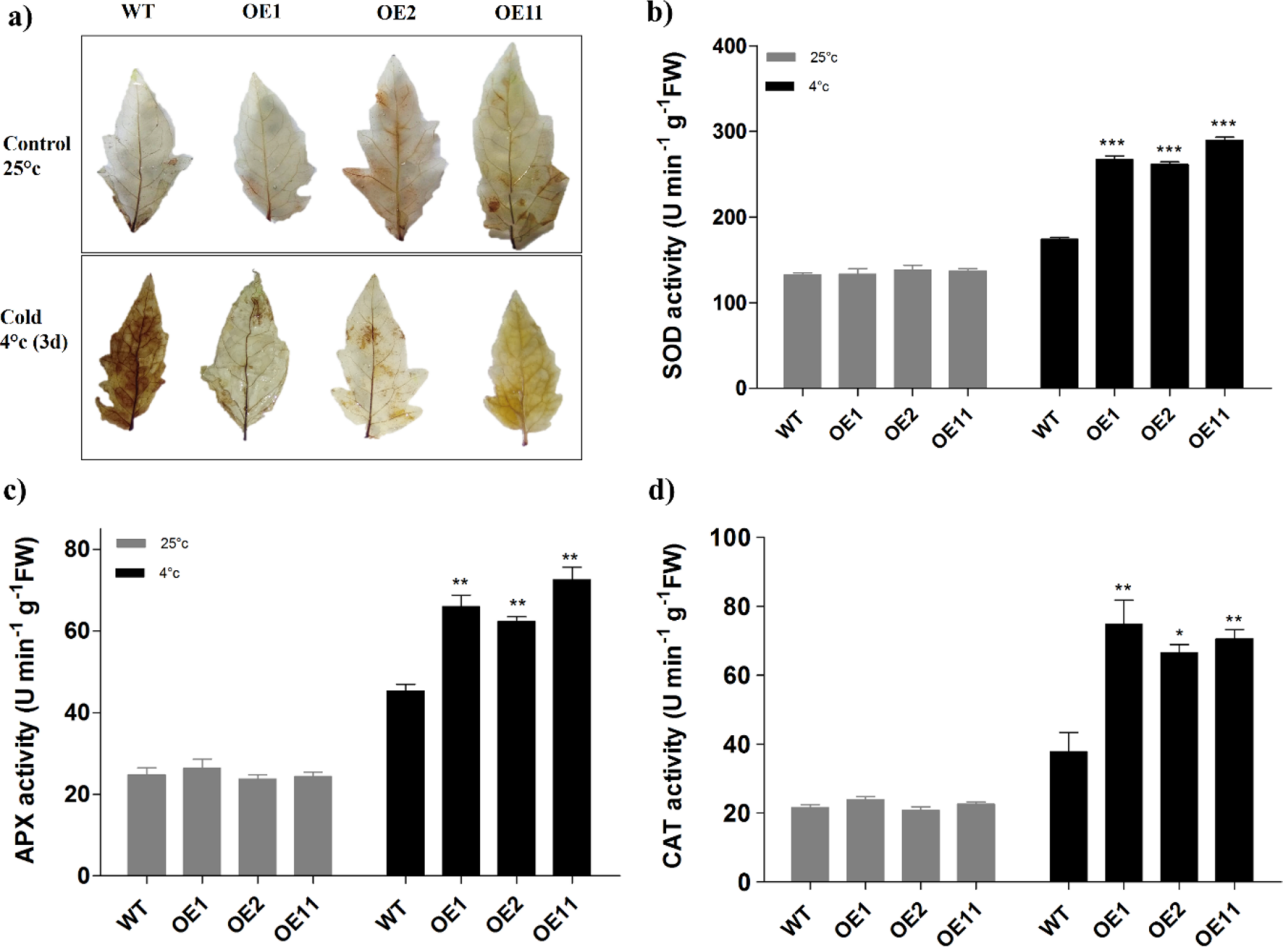
Comparison of ROS accumulation and antioxidant enzymatic activity in wild-type and *BoCRP1* over-expressing transgenic lines under cold stress. (**a**) DAB assay of 6 week old seedlings depicting staining of H_2_O_2_ with DAB in WT and transgenic leaves under Normal (25 °C) and cold stress (4 °C for 3 days). The leaflets with dark brown regions shows H_2_O_2_ accumulations (**b–d**) represents the comparison of antioxidant enzyme activity of SOD, APX and CAT in wild-type and transgenic lines under both normal and cold stress. The data represents the mean of three independent biological replicate samples. The bars indicates standard deviation (SD) and asterisks represents the significant differences between wild-type and transgenic lines at *P < 0.05; **P < 0.01; ***P < 0.001 Tukey's Multiple Comparison Test.

To evaluate the extent of ROS accumulation under cold and normal temperatures in both WT and *BoCRP1* transgenic lines, hydrogen peroxide (H_2_O_2_) staining of leaves was carried out. The staining pattern was almost similar in WT and transgenic leaves grown under normal temperature (25 °C). However, after 3 days of cold treatment (4 °C), we observed significantly higher staining (dark brown spots) in WT plants relative to transgenic lines (Fig. 6a). The reduced staining pattern observed in transgenic lines depicts improved detoxification of H_2_O_2_ in the transgenic lines. These attributes were well linked with reduced levels of REL and MDA content in *BoCRP1* expression lines, indicating reduced oxidative damage under cold stress. Under cold treatment, the *BoCRP1* transgenic lines showed nearly 2.1, 3.0, and 3.1 fold increase in SOD, APX and CAT activity respectively compared to 1.8, 1.3 and 1.7 fold increase of these genes in WT maintained at 25 °C (Fig. 6b–d). These results suggest that *BoCRP1* overexpression led to decrease in the levels of MDA and enhanced antioxidant capacity resulting in reduced oxidative injury to the transgenic tomato plants.

### *BoCRP1* over-expression enhanced Stress Responsive genes in transgenic tomato

**Figure 7:**
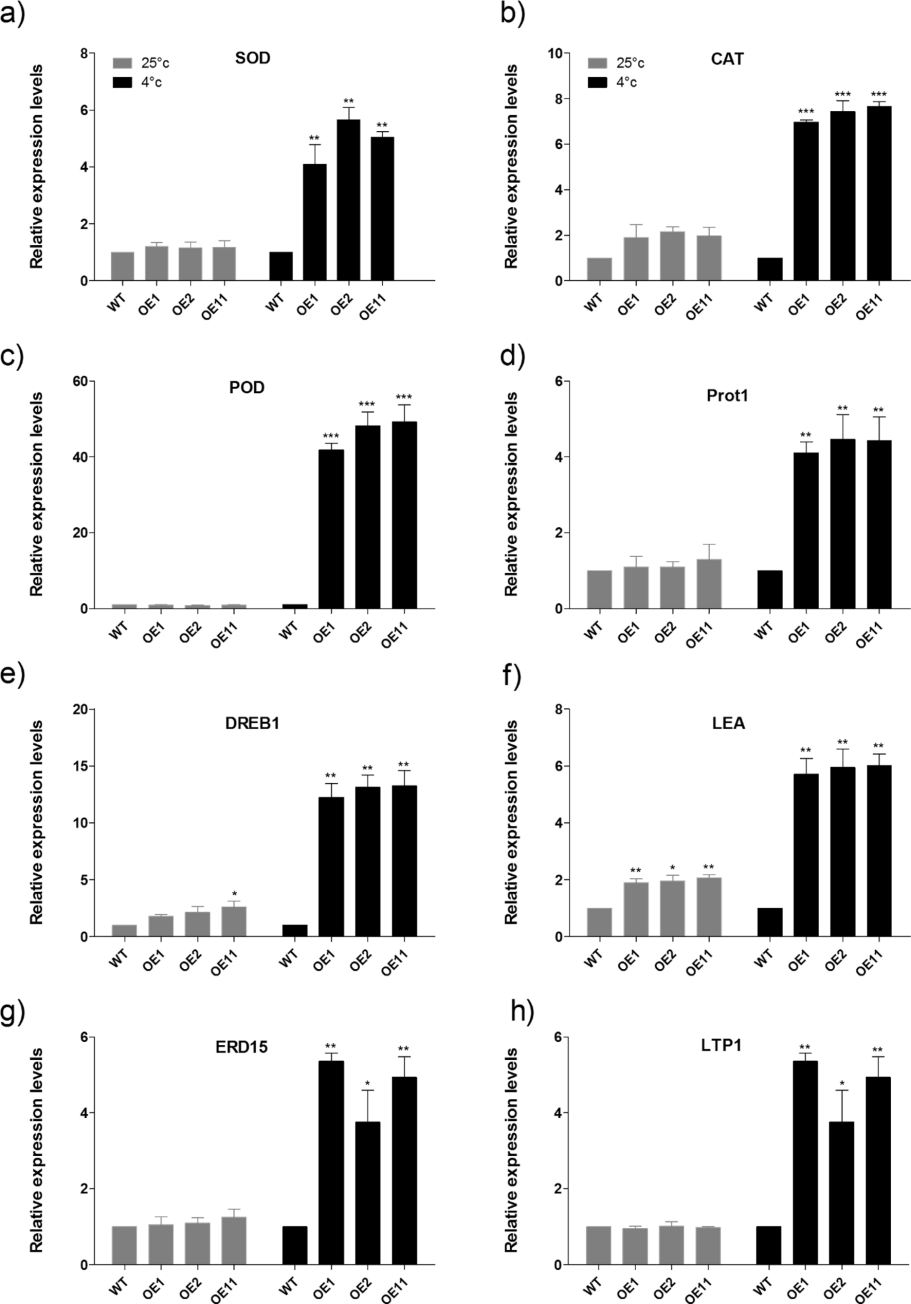
Expression levels of stress responsive genes in wild-type and *Bo*CRP1 transgenic lines. (**a–h**) depicts the transcript levels of SOD, POD, CAT, ProT1, DREB1, LTP1, ERD15-2 and LEA in wild-type and BoCRP1 overexpression lines. Six week old seedlings of transgenic lines and wild-type plants treated with cold (4 °C) or 25 °C (control) for 3 days were used to collect the samples for RNA extraction. For relative expression levels, 2^−∆∆CT^ method was employed using β-Tubulin as internal control to normalize the expression levels of target genes. Data represents the mean ± SD of three biological replicates. Each sample replicate composite of leaves was collected from a total of 12 seedlings. WT represents the wild-type tomato, OE1, OE2 and OE3 represent three independent putative BoCRP1 transgenic tomato lines. Asterisks represents the significant differences between wild-type and transgenic lines. *P < 0.05; **P < 0.01, ***P < 0.001 Tukey's Multiple Comparison Test.

To further widen our understanding of the molecular mechanism that governs the enhanced tolerance to cold stress in *BoCRP1* transgenic lines, we analysed the mRNA expression levels of eight ROS associated/stress response genes in both control as well as transgenic lines maintained under cold stress. Following the exposure to cold treatment (4 °C) for 3 days, the mRNA levels of ROS detoxification enzymes (POD, CAT and Cu–Zn SOD), significant regulatory protein (DREB1), proline Transporter 1(ProT1), Lipid transfer protein(LTP1) and stress defensive proteins (EDR15-2 and LEA) was significantly up-regulated *BoCRP1* expressing lines compared to WT (Fig. 7a–h). These findings therefore, led to the conclusion that the enhanced stress responsive in *BoCRP1* lines is a consequence of elevated expression of stress-associated genes.

## Discussion

The KIN genes are low molecular weight proteins that belong to the COR gene family and serve to offer protective functions to the plant against cold stress. Two of the KIN proteins are arranged tandemly in the genome of *A. thaliana* whose expression has been seen to go up when exposed to NaCl, ABA, polyethylene glycol (PEG) and cold^28–30^. Many homologues of KIN genes such as BN28a and BN28b, responsive to cold stress, have been identified in *Brassica napus*^31,32^. However, there are still many missing links that need to work so as to offer clear insights into the molecular and physiological mechanism of Kin gene expression and establish its role in stress-responsive mechanism. In this study, we have identified a novel cold-resistant protein1 (*BoCRP1*) from different varieties *B.oleracea* like *acephala* (Kale), *capitata*(cabbage), *brotrytis*(cauliflower) which was found to be homologous with other KIN proteins from *A. thaliana*, *B. rapa* and *B. napus*, annotated as cold-resistant proteins (Fig. 1a,b)^26^. After analysing the expression of *BoCRP1* at mRNA and protein level under both normal and cold conditions, we observed that the expression remained more or less constitutive under normal temperatures and got highly induced under cold (Fig. 2b). Furthermore, the comparison of *BoCRP1* transcript in different tissue types such as leaves root, and stem revealed higher expression of *BoCRP1* in leaves compared to root and stem under normal and stress conditions with enhanced expression under cold treatment (Fig. 2a). This is consistent with what has been observed for other Kin family members such as BN28 of *Brassica napus*^32^ and BoKIN1 of *B. oleracea*^33^ and is also consistent with its proposed role in the process of cold acclimation, wherein the leaf seems to bear the major brunt of cold stress compared to root and stem. These results are therefore suggestive that *BoCRP1* have role in imparting cold tolerance that was further validated through its functional characterization in cold susceptible tomato.

To explore the physiological and molecular involvement of *BoCRP1* in cold stress tolerance, we generated *BoCRP1* expressing lines via transformation following the protocol of Arshad et al. ^34,35^. Our findings showed that *BoCRP1* over-expression significantly improves the cold tolerance in transgenic tomato along with higher germination rate (Fig. 4a,b), increased root and hypocotyl length relative to WT when exposed to cold stress (Fig. 4c–e). Moreover, compared to wild-type, transgenic lines OE1, OE2 and OE11 displayed a marked increase in survival rate when subjected to cold stress for 4 days and returned to 25 °C (Fig. 4f,g) suggesting a protective role of *BoCRP1* against cold stress in tomato.

Under abiotic stress, the accumulation of the two major osmolytes, soluble sugar and proline^36–38^ was observed to improve the osmotic potential by retaining the water in the cells and reduce any loss of water, which prevents the disruption of cellular metabolism. It has been found that the proline brings about the activation of some stress-responsive genes^39^ and hence relaying its osmoprotective function by causing enhanced expression of stress related genes. Besides relying on the osmoprotectants, plants also utilize some soluble sugars as a nutrient during stress conditions^40^. We also obtained an increase in accumulation of soluble sugars and proline content only in *BoCRP1* expressing lines when exposed to cold stress (Fig. 5a,b). A number of compatible solutes have been reported so far with an ability to confer tolerance against cold stress with proline and soluble sugars playing important role in rendering the plant cold tolerant^41^.

The above data was corroborated by the finding suggesting an enhanced expression of ProT1 transcript in the transgenic lines relative to the WT under cold conditions (Fig. 7d). The ProT1 gene is implicated to have a role in the biosynthesis of proline^42,43^ suggesting that transgenic lines expressing *BoCRP1* suffered minimum damage due to reduced loss of intracellular water, partially by accumulating the elevated levels of intracellular osmolytes^44,45^. Moreover, in transgenic lines OE1, OE2 and OE11 the transcript levels of LEA protein was significantly up-regulated under cold stress compared to wild-type tomato plants. The increased expression of LEA proteins under abiotic stress alleviates the osmotic stress and protects the plant from dehydration^38,46^.

The plant cells under cold stress frequently generate and accumulate a huge load of free radicals that have a damaging effect on the plant cell membranes that may ultimately result in cell death^47,48^. Under cold stress conditions, the plant cellular membranes gets damaged to a large extent which is clearly reflected by intracellular levels of REL and MDA^49,50^. The MDA and REL are indirect but important indicators for evaluating the response of a plant to cold stress as both can serve as markers of the extent of damage that a membrane has suffered^51^. In the study, it was observed that in *BoCRP1* expressing lines, the level of MDA and REL were significantly lower compared to wild-type plants(Fig. 5c,d) hence, strongly suggesting a pivotal role of *BoCRP1* protein in protecting the plant cells from lipid peroxidation. In *A. thaliana* lines expressing Cor15a in increased amount, tolerance to cold has been observed as there is a marked decrease in freezing induced membrane dehydration in protoplasts when exposed to chilling temperatures^52^.

Plants are well equipped with an effective ROS scavenging defence mechanism that include enzymatic antioxidants such as Ascorbate peroxidase (APX) ,catalase (CAT), superoxide dismutase (SOD) and peroxidase (POD) and some non-enzymatic antioxidants which protect the cellular structures and other macromolecules from damage caused ROS molecules^53,54^. We have observed that the hydrogen peroxide (H_2_O_2_) levels, as depicted in Fig. 6a were significantly higher in WT under chilling stress compared to *BoCRP1* expressing lines. The proteomic investigation by Xu et al.^55^ also reported a huge spike in ROS levels and death of leaf cells in frost-sensitive winter wheat cultivars when they were exposed to compared to the frost-tolerant cultivar leaves that accumulated significant amount of antioxidant-related proteins. In the present study, we observed *BoCRP1* expressing lines displayed increased activity of SOD, APX and CAT compared to the WT (Fig. 6b–d) and that could be attributed to the resilience of transgenic tomato lines to cold stress as compared to WT. The SOD acts as a scavenging enzyme by acting upon superoxide and convert it to H_2_O_2._ Consequently, the APX and CAT act on H_2_O_2_ and perform its detoxification^26^. The higher accumulation of ROS scavenging enzymes in *BoCRP1* expression lines is concomitant with reduced accumulation of H_2_O_2_ in transgenic lines as depicted in DAB assay.

Based on the above results**,** we can assume that enhanced expression of *BoCRP1* imparted cold tolerance in tomato possibly by activation of genes involved in generating a stress response. To investigate this, we performed qPCR analysis of some stress associated genes including the ROS detoxifying enzymes (SOD, POD, and CAT), lipid transfer protein 1 (LTP1), Dehydration response element-binding protein (DREB1), Proline biosynthesis gene (ProT1) and Late embryogenesis abundant proteins (ERD15-2 and LEA). The quantitative analysis of these stress-responsive genes led to several interesting findings. SOD, CAT and POD involved in ROS detoxification mechanism are highly induced under cold in the transgenic lines relative to Wild-type plants (Fig. 7a–c). The observations are in consonance with the increased activity of these enzymes that results in reduced accumulation of H_2_O_2_ in *BoCRP1* expressing lines on exposure to cold. Furthermore, the up-regulated expression of LTP1 in transgenic lines relative to the WT plants (Fig. 7f) also exhibited consistently reduced MDA and REL. Since LTP1 is involved in lipid metabolism^56^ it’s up-reregulation in *BoCRP1* expressing lines under chilling stress indicates its role in reducing the damage to lipids and membranes. ProT1 which is involved in proline biosynthesis showed enhanced expression under chilling stress in transgenic lines than WT (Fig. 7d) which is concomitant with the enhanced accumulation of Proline in BoCRP1 expressing lines under cold. DREB1 (significant regulatory protein) along with EDR15-2 and LEA (stress defensive proteins) showed increased expression under cold stress in over-expression lines compared to WT (Fig. 7e–h). These stress-responsive genes protect the plant cells against different types of environmental stresses by stabilizing the liable enzymes, protecting macromolecules and cellular membranes^57^. This further explains the reduced accumulation of REL and MDA content and increased survival of transgenic lines under chilling stress. Our results are in complete concordance with the study carried out by Liu et al.^38^, Munir et al.^44^, Liu et al.^56^ with regard to the up-regulation of stress responsive gene under abiotic stress. Based on the results discussed, we can conclude that the *BoCRP1* acts as a multifunctional protein that acts through the downstream proteins to combat the cold response in plants. To further investigate the *BoCRP1*mechanism in modulating the cold tolerance, we need to unravel its regulation and downstream target proteins.

## Materials and methods

### Plant material

Seeds of *Brassica oleracea* var “Capitata” and *Lycopersicum esculentum* var “shalimar 1” collected from SKUAST Kashmir were used in the present study after obtaining permission from institutional ethical committee and local Biodiversity Board. All the experiments were carried in accordance with national and international guidelines. Seeds of *B. oleracea var. “Capitata”* were sown in a mixture of vermiculite, peat moss and soil prepared in the ratio of 1:2:1 and were allowed to grow in the greenhouse with a photoperiod cycle of 16/8 h (day/night) for six weeks. The plants, when required were given cold stress in a controlled cold chamber maintained at 4 °C for a maximum period of 3 days. At different intervals samples were collected and stored at − 80 °C. Seeds of *L. esculentum* var*.* “Shalimar 1” were thoroughly rinsed in sterile distilled water for about 10 min followed by surface disinfection with 70% (v/v) ethanol for 3-min. The seeds were then washed for 15 min in a solution containing sodium hypochlorite (4% (v/v)) and few drops of Tween-20 and rinsed 3 times with sterile water for 3 min and allowed to germinate on solidified MS medium with half strength^58^, mixed with 15 g L^−1^ sucrose with a resultant pH of 5.8 and the plates were maintained at 25 °C in the dark for 2 days. Post germination the seedlings were grown at 25 °C for 7–10 days maintained at 16/8 h (day/night). Cotyledonary explants of 10 day old seedlings were cut from proximal as well as on distal sides and cultured on pre-culturing medium for 2 days followed by co-cultivation with *Agrobacterium* in dark for 3 days. Furthermore, to check the combined effect of plant growth regulators, we carried out three independent experiments containing nearly 150 explants in each experiment**.**

### Isolation of BOCRP1 and multiple sequence alignment

Total RNA was isolated from B oleracea leaf sample using trizol reagent (Invitrogen). To synthesize the first- strand cDNA, Super Script™ VILO™ (Invitrogen) was used as per the manufacturers protocol. The full-length open reading frame (ORF) was amplified from leaf cDNA of *B. oleracea* using *BoCRP1* specific primers (Gene Bank accession no. GQ461800.1). To clone the amplified fragment, we used pGEMT Easy Vector Systems (Promega) and consequently the clones were sequenced for identification of desired sequence. A tblastp search was executed on http://www.ncbi.nih.gov.edu and the proteins with low E-values were obtained from the sequence database. A multiple sequence alignment of the obtained sequences was executed using ClustalX to find domains conserved across the sequences. A phylogenetic tree was also constructed by the neighbor-joining method to determine how these aligned proteins are related to each other.

### Cloning of *BoCRP1* in plant binary vector and *Agrobacterium*-mediated transformation of tomato plants

To construct *BoCRP1* recombinant vector, the coding sequence of *BoCRP1* (cold resistant protein 1) accession number GQ461800.1 was amplified from *B.oleracea* and cloned into *Bam*H1 and *Sac*I site downstream of stress inducible AtRd29A in PCAMBIA2301 plasmid (Fig. 3a). The recombinant vector harbouring the *BoCRP1*gene was introduced into super-virulent strain of *Agrobacterium,* GV3101 using the freeze–thaw method. Transformation of recombinant vector was confirmed through PCR and restriction digestion. 10 days old cotyledons of tomato seedlings were co-cultivated with agrobacterium and transformed cotyledons were selected on Kanamycin positive media (50 mg L^−1^). After selection, the transformants were regenerated on shoot regeneration medium (SRM) containing MS agar mixed with 2 mg L^−1^ zeatin, 0.1 mg L^−1^ IAA and 250 mg L^−1^ cefotaxime and 50 mg L^−1^ kanamycin. Regenerated shoots were transferred to the root regeneration medium (RRM) and hardened in a mixture containing 1:1 ratio of soil and vermiculite in the PVC pots and maintained in greenhouse at Kashmir University Botanical Garden.

### Validation of *BoCRP1* expressing transgenic lines through PCR

Genomic DNA was isolated from the leaf tissues by CTAB method^59^. The transgenic lines were confirmed by performing PCR using primers specific to NPTII and *BoCRP1*. The PCR reaction was performed using 100 ng of genomic DNA as template, 1 × PCR buffer (MgCl_2_ included), 0.25 mM dNTP mix (sigma), 0.25 µm forward and reverse primers and 0.05 U Taq DNA polymerase (sigma) with a reaction volume of 20 µl The reaction conditions were set with an initial denaturation(95 °C) for 10 min, 35 cycles of following reaction parameters: 95 °C/1 min, 60 °C/30 s, 72 °C/ 40 s with final extension of 72 °C for 10 min. The amplified fragments were confirmed on 1% agarose gel and visualized under a UV illuminator**.**

### Evaluation of transgenic tomato lines using Southern blot

For copy number insertion, we performed Southern blotting as per the protocol published by Southern^60^. Initially 15 µg of genomic DNA was digested with *Bam*H1 restriction enzyme and allowed to run overnight on 0.8% agarose gel. The DNA was then transferred to Amersham Hybond-N^+^ membrane (manufactured by GE Healthcare). This was followed by cross-linking of DNA with membrane by exposing the membrane to UV (1200 µJ for 5 min) as explained by Russell and Sambrook^61^. These membranes with cross linked DNA fragments were hybridized with probe directed against NPTII that was labelled with dioxigenin using DNA labelling kit (Roach Sigma) and later exposed to X-ray film (Amersham Hyperfilm ECL GE Healthcare). Hybridization, membrane washing, DNA probe preparation, were performed as per the mentioned protocol (DIG Nonradioactive Labelling and Detection system Roch).

### qPCR analysis

For the quantitative analysis, total RNA was extracted from the leaf tissue of wild-type and transgenic tomato plants by TRIZOL reagent (Invitrogen) and subjected to DNase I (New ENGLAND BIOLABS). After checking the integrity of RNA, nearly 2 µg of purified RNA was used to synthesize the first strand cDNA using Super Script VILO cDNA Synthesis Kit (Invitrogen). qPCR analysis were performed to detect the transcript levels of *BoCRP1* across different transgenic and wild-type plants that were either exposed to cold stress (4 °C) or maintained at normal temperature conditions (25 °C). qPCR was also performed to analyse the transcripts corresponding to both ROS related as well as stress responsive genes in WT and the transgenic lines. In order to calculate the relative expression levels, 2^−∆∆CT^ method was employed^62^ using β-Tubulin as internal control to normalize the expression levels of target genes. Both the real-time and full length primers were designed using primer-3 online bio-informatics tool. The primers designed were also crossed checked using Gene Runner tool. For qPCR analysis primer efficiency was calculated for each primer set using tenfold dilution method. In each experiment three biological replicates were used.

### Protein extraction from plant tissue and Western blotting

Frozen leaves of transgenic and wild type tomato were ground to a fine powder using a pre-chilled mortar and pestle. The frozen sample powder was transferred to ice-cold extraction buffer (0.2 M 3-(N-morpholino) propanesulfonic acid (MOPS), pH 7; 0.5% (w/w) polyvinyl polypyrrolidone; 1% (v/v) Triton X-100; 10% (v/v) glycerol; 2 mM dithiothreitol (DTT) and proteinase inhibitor cocktail) and ground further with a chilled mortar and pestle. The extract obtained was filtered through a 20 to 70 µm Miracloth, squeezed by hand to remove cell walls and other debris and centrifuged for 15- 20 min at 30,000 × g at 4 °C. Supernatant was carefully decanted and 5X SDS sample buffer was added to it. The mixture was boiled for 5 min at 100 °C and resolved by SDS-PAGE. Proteins were transferred on PVDF membrane, probed with in house anti-CRP1 antibody followed by washings with PBS-tween and final incubation in IR-tagged anti-mouse secondary antibody. Visualization was carried out on IR scanner (Licor Biosciencs).

### Evaluation of response of transgenic tomato plants to cold stress

#### Germination and seedling growth assay

To determine the effect of cold stress on germination, seeds from homozygous T3 transgenic lines (OE1, OE2 and OE11) and WT were allowed to germinate on MS media. For evaluating the cold stress tolerance, approximately 26 seeds were plated on MS media and placed in the controlled growth chamber which was maintained at 25/4 °C with 16/8 h (day/night) light cycle. The rate of seed germination were evaluated after a period of one week. For the seedling growth assay, the transgenic and wild-type lines after germination were transferred to 10 °C for nearly two weeks. The main root length was measured with a ruler. Pictures were taken after treatment at 10 °C. Each experiment was repeated thrice.

### Estimation of malondialdehyde (MDA), relative electrolyte leakage (REL), proline and total soluble sugars

Electrolyte leakage (REL) was evaluated using the protocol described by Hu et al.^63^ with certain modifications. For this the leaves were excised into fine strips and incubated at 28 °C in 10 mL of distilled water for approximately 8 h. we measured the initial conductivity(C1) using the conductivity meter (Systronics, India). The samples were boiled in a water bath for 10 min and then cooled to room temperature to measure the electrolyte conductivity (C2). The extent of REL was evaluated using the equation: EL (%) = C1/C2 × 100. The levels of Proline was estimated by Bates et al.^64^. Leaf tissue (0.5 g) harvested from wild-type and transgenic lines under normal and cold conditions were ground in 10 ml of 3% (w/v) sulfosalicylic acid aqueous solutions. For further analysis the homogenate was filtered through Whatman no. 1 filter paper. 2 ml of acid ninhydrin (1.25 g ninhydrin were dissolved in a mixture of 30 ml glacial acetic acid and 20 ml of 6 M phosphoric acid) were combined with 2 ml of filtered solution and incubated at 100 °C for 1 h. The reaction was terminated on ice bath, this was followed by addition of 4 ml of toluene to the reaction mixture and organic phase was warmed to ambient temperature and its optical density was measured in UV–VIS spectrophotometer (Shimadzu, Japan) at 520 nm using toluene as blank. The total amount of proline was subsequently determined from a standard curve. Malondialdehyde were assessed according to protocol described by Choudhury et al.^65^. while the content of soluble sugar was determined following the outline of SY, Chen et al.^66^ For estimation of total soluble sugar, leaves were incubated at 80 °C in 80% ethanol for 30 min with occasional agitation. For measuring the total soluble sugar content, 5 ml of anthrone reagent was added to 1 ml filtered extract and incubated at 95 °C for 15 min. The reaction was terminated in ice bath and absorbance was measured at 620 nm.

### Histochemical detection of H_2_O_2_

The relative content of H_2_O_2_ was detected visually in both transgenic as well as WT tomato leaves by utilizing 3, 39-diaminobenzidine (DAB) staining method described by Bindschedler et al.^67^. The leaves were collected after 3 days of cold stress at 4 °C and incubated with 1 mg ml^−1^ DAB containing 10 mM sodium phosphate buffer pH 7.0 and 0.05% v/v Tween 20 for 6–7 h. when brown precipitate starts visible in wild-type plants, the leaves were boiled in 3:1:1 ratio of ethanol: acetic acid: glycerol for 10–15 min or until all the chlorophyll was completely bleached.

### Determination of enzymatic antioxidants

For evaluating the activities of various antioxidant enzymes such as SOD, APX, and CAT, we followed the protocol outlined by Liu et al.^56^. Leaf sample approximately 200 mg was homogenized in 2 ml of ice cold 0.1 M phosphate buffer (pH 7.0) and were centrifuged for 15 min at 13,500×g. The supernatant was collected and used for determination of SOD, APX, and CAT enzyme activities. The activity of SOD was measured spectrophotometrically at 550 nm by observing inhibition of the cytochrome c reduction rate in the presence of the xanthine–xanthine oxidase. At 25 °C, one unit of activity is defined as the quantity of enzyme that limits the rate of cytochrome c reduction by 50%. According to Mittova et al., APX activity was evaluated by measuring the oxidation rate of ascorbate at 290 nm^68^. The activity of CAT was monitored by measuring H_2_O_2_ breakdown at 240 nm using UV–VIS spectrophotometer (Shimadzu, Japan)^68^.

### Statistical analysis

The data was statistically analyzed using GraphPad Prism 5. Each experimental data represents the mean ± SD of three Biological replicates such that each sample from the replicate was a combination of leaves corresponding to 10 different seedlings. Tukey's Multiple Comparison Test were performed to calculate significant differences in transgenic lines compared to WT. Asterisks represents significant differences at *P < 0.05; **P < 0.01; ***P < 0.001.

## Conclusion

As a whole, our findings indicates that the over-expression of *BoCRP1* increased the adaptability of tomato plants to cold stress. Besides there are many other manifestations of cold tolerance such as minimalistic damage to membrane with increased scavenging potential of antioxidant enzymes such as CAT SOD, POD and APX, reduced accumulation of MDA, REL and H_2_O_2,_ enhanced expression of genes involved in stress and increased accumulation of osmoprotectants (proline and soluble sugars). This study would help us in gaining new insights for manipulating the chilling tolerance in cold susceptible crop plants without hampering the overall growth and development of the plant.

## Supplementary Information

Supplementary Information.
