# Supplementary material for: Ectopic expression of a novel cold-resistance protein 1 from Brassica oleracea promotes tolerance to chilling stress in transgenic tomato
Source: Sci Rep. 2021 Aug 16;11:16574. doi: 10.1038/s41598-021-96102-3 (PMC8367951; doi:10.1038/s41598-021-96102-3)
Supplement: Supplementary file 1 — Supplementary Information. [file 41598_2021_96102_MOESM1_ESM.docx]

**Supplementary data**

**Ectopic expression of a novel cold-resistance protein 1 from *Brassica oleracea* promotes tolerance to chilling stress in transgenic tomato.**

Umer Majeed wani^1,2^ Sheikh Tahir Majeed^2^, Vaseem Raja^1^, Zubair Ahmad Wani^2^, Nelofer Jan^1^, Khursid Iqbal Andrabi^2^, Riffat` John^1*^

^1^ Plant Molecular Biology Department of Botany, University of Kashmir.

^2^ Department of Biotechnology, University of Kashmir.

**
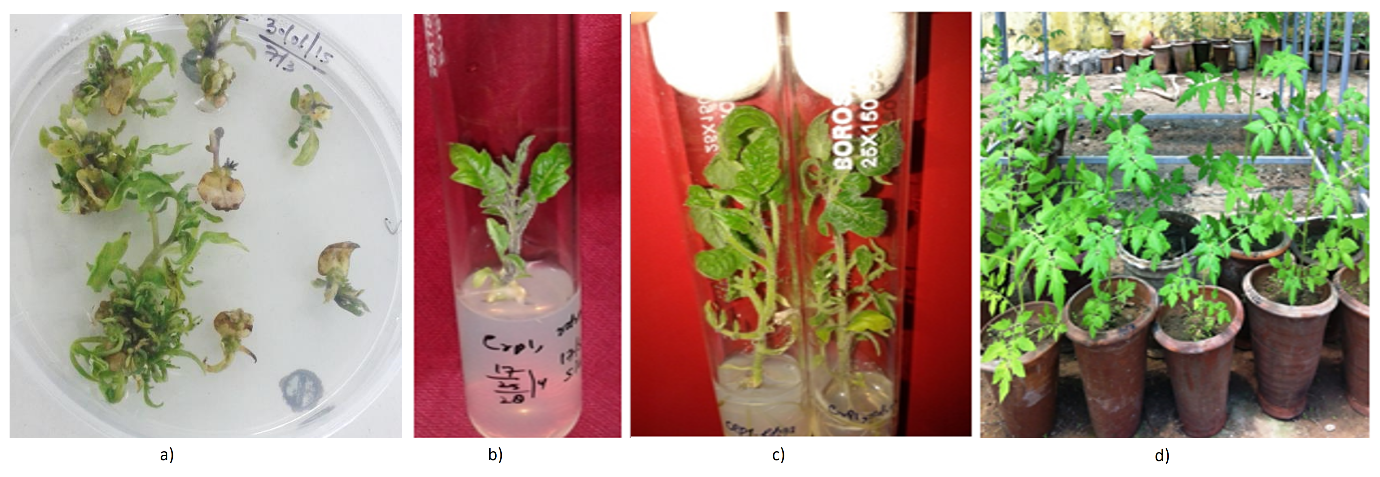
**

**Figure S1. Generation of transgenic lines via agrobacterium mediated transformation of tomato plants**. **(a)** Depicts the transgenic shoot lets generated transformed cotyledons on selection media containing kanamycin as selection marker along with Zeatin and IAA **(b)&(c)** transgenic shoots generated from each cotyledon were individually shifted on shoot elongation medium containing 50 mg L^-1^ kanamycin , 2 mg L^-1^ zeatin, 0.1 mg L^-1^ IAA and then shifted to root elongation medium containing 50 mg L^-1^ kanamycin , 0.5 mg L^-1^ zeatin, 1 mg L^-1^ IAA to generated full-fledged transgenic plants with developed root system. **(d)** The transgenic plants were moved from test tubes to pots containing mixture of soil and vermiculite. In order to adopt to the external environment and maintain under high humidity these pots were kept under polythene for few days before exposed to greenhouse conditions.

**Figure 3b**

**
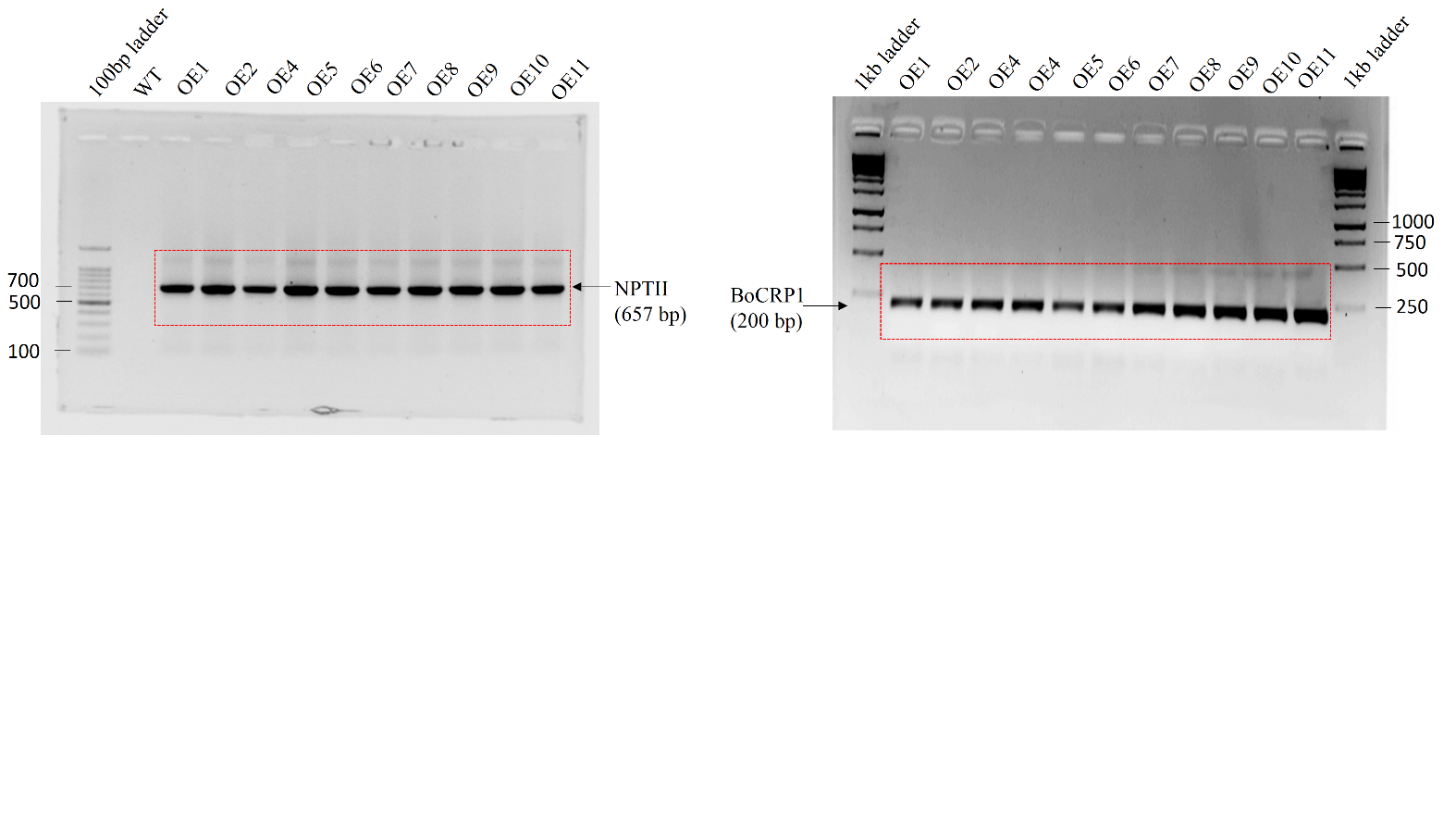
**

**Figure 3C**

**
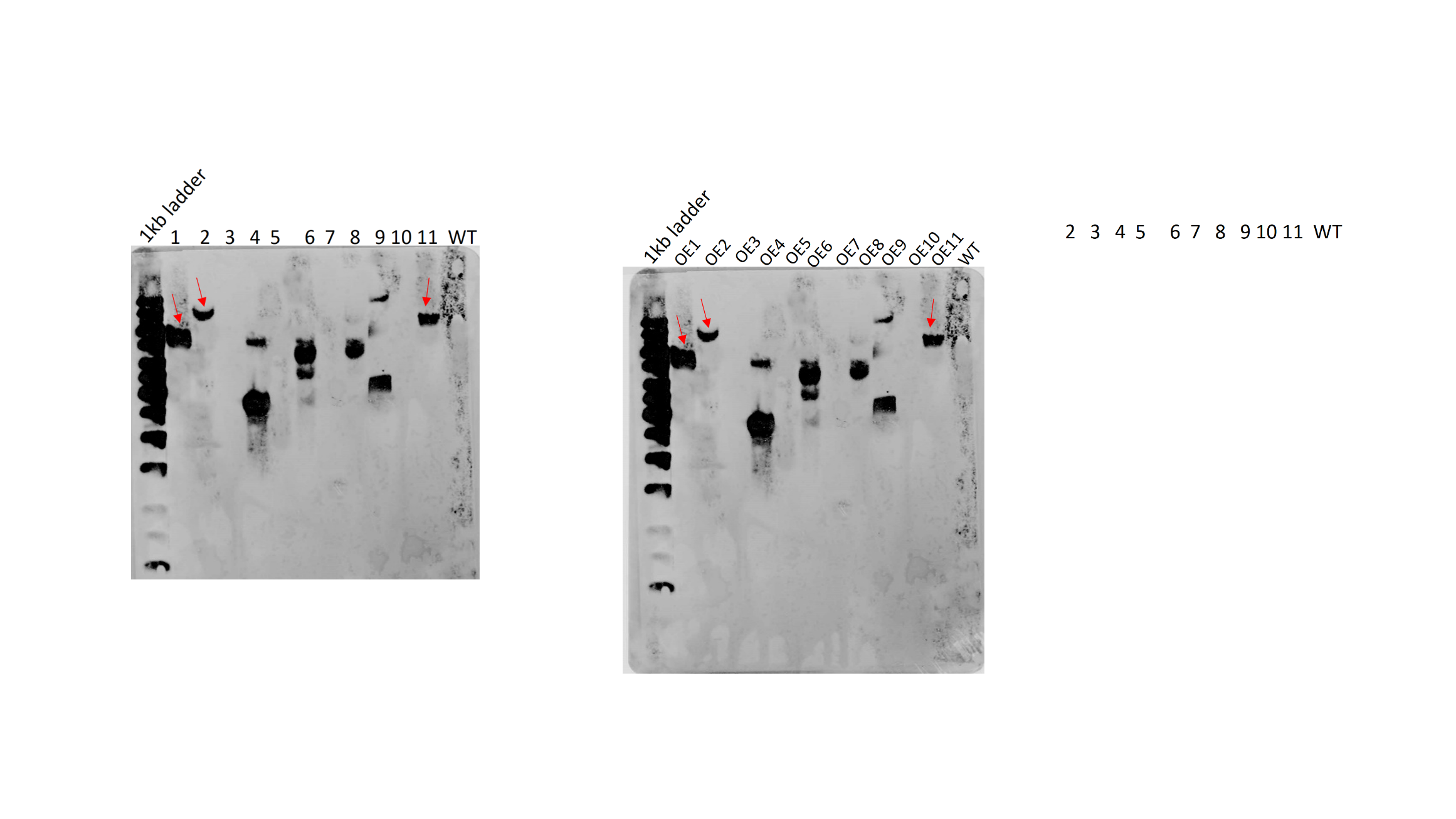
**

**Figure 3e**

**
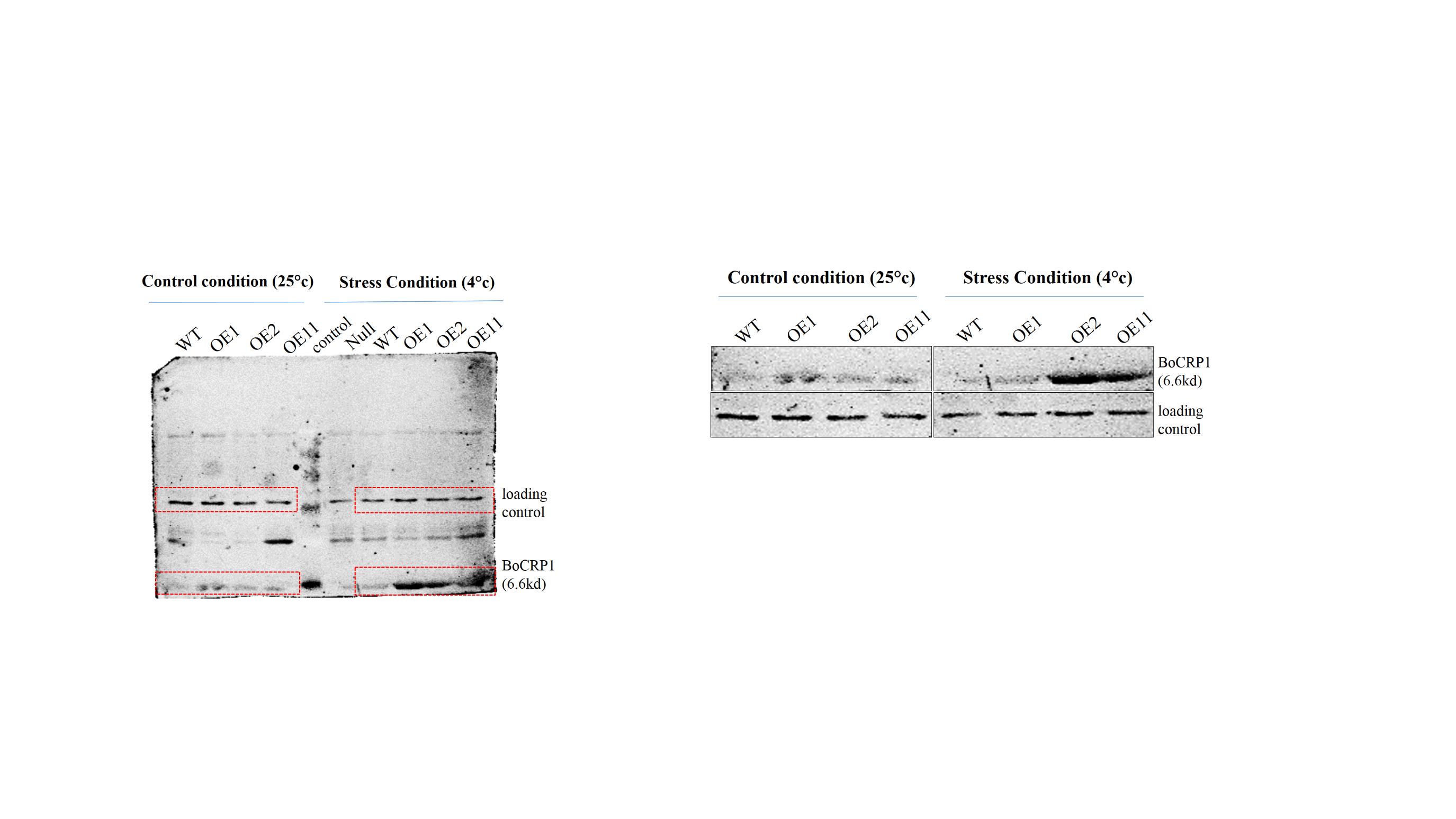
**

**Figure S2**. Uncropped Gels/Immunoblots of Figure 3 depicting cropped area (red dotted line)**.**

**Table S1: primers used in cloning and qPCR analyis**

| **Gene Name** | **Forward primer** | **Reverse primer** |
| --- | --- | --- |
| BoCRP1 | GCTGGATCCATGGCAGACAACAAGCAGAGCTTCC | GACGAGCTCCTACTTGATCATGCCGGTCTTCTCC |
| rd29A promoter | GCGAAGCTTCGACTCAAAACAAACT TACGAA | GCTGGATCCAATCAAACCCTTTATTCCTGATG |
| NPTII | TAAAGCACGAGGAAGCGGTC | ATTCGGCTATGACTGGGCAC |
| SOD | CTCCTGGACTTCACGGGTTT | TGAACCACAAGTGCTCGTCC |
| POD | CCTTGTTGGTGGGCAC ACAA | GGCCACCAGTGGAG TTGAAA |
| CAT | TCCCAGTTGGTCGATTGGTG | GATGACCACACTTGGGAGCA |
| ProT1 | ACCGCCCATCAAGTTAGCAA | CCTCTTGCCCCCAACTTCAT |
| ERD15 | CAAGGAATCAAGTCAT CTCTCTATG | TCTAATCAGTGCAT CAGATGGT |
| DREB1 | GGTTTGTGTGCCGTTGGATT | CACCGCCAATTTTCTGGTCC |
| LEA | CAATTCCCTTTCGTTCCAGA | TCTTCCTGATGCGATTACCC |
| LTP1 | TCTAGGAGGCTGTTGT GGTG | GTGGAGGGGCTGAT CTTGTA |
| Βeta-Tubilin | TCCTAGGGCGGTTTTGATGG | AGTGGCAAACCTGGAATCCT |
